# Clinical Characteristics Predicting Worse Long-Term Outcomes in Patients with Myocardial Infarction and Non-Obstructive Coronary Arteries (MINOCA)

**DOI:** 10.3390/jcdd9090286

**Published:** 2022-08-26

**Authors:** Piotr Szolc, Łukasz Niewiara, Paweł Kleczyński, Krzysztof Bryniarski, Elżbieta Ostrowska-Kaim, Kornelia Szkodoń, Piotr Brzychczy, Krzysztof Żmudka, Jacek Legutko, Bartłomiej Guzik

**Affiliations:** 1 Department of Interventional Cardiology, Faculty of Medicine, Institute of Cardiology, Jagiellonian University Medical College, 31-202 Kraków, Poland; 2 Clinical Department of Interventional Cardiology, John Paul II Hospital, 31-202 Kraków, Poland; 3 Department of Emergency Medicine, Faculty of Health Sciences, Jagiellonian University Medical College, 33-332 Kraków, Poland; 4 Students’ Scientific Group at the Department of Interventional Cardiology, Jagiellonian University Medical College, John Paul II Hospital, 31-202 Kraków, Poland; 5 Students’ Scientific Group of Modern Cardiac Therapy at the Department of Interventional Cardiology, Jagiellonian University Medical College, John Paul II Hospital, 31-202 Kraków, Poland

**Keywords:** myocardial infarction, MINOCA, outcome, predicting factors

## Abstract

Non-obstructive coronary artery disease occurs in 3.5–15% of patients presenting with acute myocardial infarction. This group of patients has a poor prognosis. Identification of factors that predict worse outcomes in myocardial infarction with non-obstructive coronary arteries (MINOCA) is therefore important. Patients with a diagnosis of MINOCA (*n* = 110) were enrolled in this single-center, retrospective registry. Follow-up was performed 12, 24 and 36 months after discharge. The primary composite endpoint was defined as myocardial infarction, coronary revascularization, stroke or TIA, all-cause death, or hospital readmission due to any cardiovascular event. The mean age of the study group was 64.9 (± 13.5) years and 38.2% of patients were male. The occurrence of the primary composite endpoint was 36.4%. In a COX proportional hazards model analysis, older age (*p* = 0.027), type 2 diabetes (*p* = 0.013), history of neoplasm (*p* = 0.004), ST-segment depression (*p* = 0.018) and left bundle branch block/right bundle branch block (*p* = 0.004) by ECG on discharge, higher Gensini score (*p* = 0.022), higher intraventricular septum (*p* = 0.007) and posterior wall thickness increases (*p* = 0.001) were shown to be risk factors for primary composite endpoint occurrence. Our study revealed that several factors such as older age, type 2 diabetes, ST-segment depression and LBBB/RBBB in ECG on discharge, higher Gensini score, and myocardial hypertrophy and history of neoplasm may contribute to worse clinical outcomes in MINOCA patients.

## 1. Introduction

The mechanism of myocardium damage in patients with myocardial infarction and non-obstructive coronary arteries (MINOCA) is diverse [1]. This clinical entity characterizes by heterogeneity and has a specific impact on prognosis and different treatment strategies [2]. Cardiology organizations define MINOCA as myocardial infarction with a lack of significant lesions in coronary arteries in angiography (diameter stenosis < 50%), and these, together with coronary microcirculation impairment, may be linked to a diagnosis of MINOCA [3]. Disorders mimicking MINOCA and not directly related to the coronary circulation include, among others, myocarditis, pericarditis, prothrombotic conditions, stroke, sepsis, pulmonary embolism, kidney and heart failure, as well as other heart and large vessel diseases, causing cardiac malfunctions, such as tachyarrhythmia, heart valve defects (aortic stenosis), cardiomyopathy (including stress-cardiomyopathy) or aortic diseases [4].

A large minority (24%) of patients diagnosed with MINOCA were shown to develop major adverse cardiac events during a one-year clinical follow-up [5]. Therefore, in this group of patients, the improvement of prognosis and quality of life depends on appropriate diagnosis and therapy oriented towards the etiology and disease mechanism of MINOCA.

The European Society of Cardiology (ESC) and American Heart Association (AHA) published statements on MINOCA emphasizing the heterogeneity of this patient group [2,6]. Both statements recommend using MINOCA as a working diagnosis directly after confirming non-obstructive coronary artery disease by angiography in patients manifesting with myocardial infarction (MI) [1]. At this point, the search for underlying causes of secondary myocardial injury should be investigated [7]. Although these patients have relatively poor outcomes, there is little data on clinical factors influencing the outcomes of MINOCA patients.

Therefore, we investigated factors that may be associated with long-term outcomes in patients diagnosed with MINOCA.

## 2. Materials and Methods

**Figure 1:**
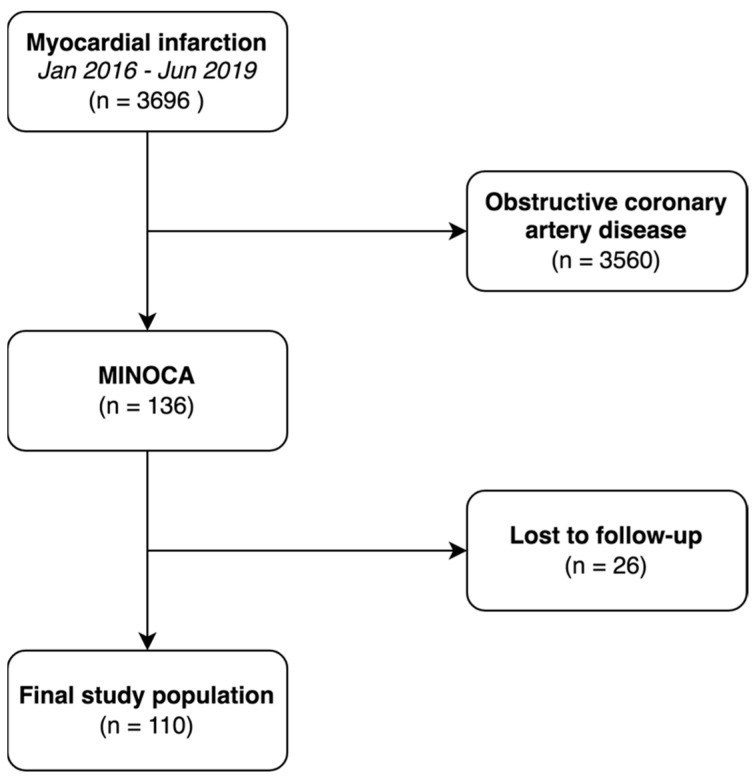
Flowchart of the study. Abbreviations: MINOCA—myocardial infarction and non-obstructive coronary arteries.

The study was conducted as a single-center, retrospective registry of consecutive patients with MINOCA. The center was a high-volume hospital with an emergency department with 24/7 percutaneous coronary intervention capability. Patients were followed-up after 12, 24 and 36 months. According to ESC guidelines, MINOCA was defined as myocardial infarction and non-obstructive coronary arteries (absence of ≥50% diameter stenosis in an epicardial artery) [8,9]. Index data were collected between January 2016 and June 2019 and were selected from 3696 patients who underwent coronary angiography due to acute MI. Of these, 136 patients were diagnosed with MINOCA. During the study, 26 patients were lost to follow-up, and we enrolled 110 adult patients (STEMI: n = 24 and NSTEMI: n = 86) with no significant lesions in epicardial coronary arteries (<50% diameter stenosis in an epicardial artery). In some cases, additional data on intravascular ultrasound and optical coherence tomography were also available. Elimination of patients from the suspected MINOCA group who, ultimately did not have a diagnosis of MINOCA (e.g., myocarditis or stress cardiomyopathy) was carried out. Patients were divided into two groups: event present and event free group. Our study did not receive any external funding. The study protocol complied with the Declaration of Helsinki and was approved by the Jagiellonian University Medical College Ethics Committee (application No. 1072.6120.279.2018). Study flowchart is presented in Figure 1.

Patients were diagnosed with acute MI according to the 4th Universal Definition of Myocardial Infarction [10]. Patients with ST-elevation myocardial infarction (STEMI) had coronary angiography performed and percutaneous angioplasty (PCI) if indicated in compliance with the European Guidelines [8]. Urgency of coronary angiography in patients with non-ST-elevation myocardial infarction (NSTEMI) was set according to their estimated risk. Patients with obstructive coronary artery disease were either referred for revascularization (angioplasty or coronary artery bypass graft) or conservative treatment according to guidelines. Further diagnostic tests such as chest computed tomography, cardiac magnetic resonance, 24 h ECG registration, and transesophageal echo, etc. were performed in patients with MINOCA according to clinical presentation and the results of all routine tests performed at baseline.

### 2.1. Demographic and Laboratory Data

Patients’ demographics, medical history, laboratory tests, echocardiographic data and medication were collected and verified from in-person interviews and medical records. Body mass index (BMI) was calculated as weight (kg) divided by height squared (m^2^). Concentrations of fasting blood glucose (FBG), triglyceride (TG), total cholesterol (TC), low-density lipoprotein cholesterol (LDL-C), high-density lipoprotein cholesterol (HDL-C), creatinine, and high-sensitive C-reactive protein (hs-CRP) were tested with an automatic biochemistry analyzer. The N-terminal pro-B-type natriuretic peptide (NT-proBNP) and cardiac troponin I (TnI) values at admission were recorded.

### 2.2. Echocardiography

A two-dimensional transthoracic echocardiography was performed by a trained physician at baseline and before hospital discharge. All measurements were carried out according to the recommendations of the American Society of Echocardiography and the European Association of Echocardiography [11].

### 2.3. Angiography

Patients without obstructive coronary arteries (no lesions with ≥50% diameter stenosis on angiography) were qualified for conservative treatment and further evaluation of the clinical cause of MINOCA. The Gensini score was calculated by summation of the individual coronary segment scores [12].

### 2.4. Follow-Up

Clinical follow-up was performed at 12, 24 and 36 months. The primary composite endpoint was defined as myocardial infarction, percutaneous coronary intervention, stroke or TIA, all-cause death, or hospital readmission due to any cardiovascular event.

### 2.5. Statistical Methods

Quantitative variables were reported as numbers and percentages. Qualitative variables with normal distribution were expressed as mean values with standard deviation, or median with standard deviation. Data normality was achieved using the Kolmogorov–Smirnov and Shapiro–Wilk tests. Qualitative variables were compared using Chi-squared tests, while continuous variables were compared using the Mann–Whitney or Student’s t-test. The univariate and multivariate Cox proportional hazards model was used to calculate the hazard ratio (HR) and 95% confidence interval (CI). Survival probability (Kaplan–Meier survival estimate) was compared using the log-rank test. A value of *p* < 0.05 was established as statistically significant. Only complete data were included in regression analysis. Statistical analysis was performed using SPSS v.26 (New York, NY, USA) and R-Studio, v.1.3 (Boston, MA, USA) software.

## 3. Results

We analyzed the data from 110 consecutive patients (38.2% males) with MINOCA. The occurrence of the primary composite endpoint was observed in 40 patients. The mean age of patients was 64.9 (±13.5) years and the BMI was 27.7 (±5.44) kg/m^2^. Arterial hypertension and dyslipidemia were the most frequent risk factors. Depending on primary composite endpoint occurrence, we segregated the study participants into two subgroups, with detailed clinical characteristics presented in Table 1. The mean follow-up duration in a whole study group was 724 (±308) days. Patients in the event-positive subgroup were older compared to the control group (68.4 vs. 62.9 years, *p* = 0.04) and had higher occurrences of type 2 diabetes and a history of neoplasm (30.0% vs. 12.9%, *p* = 0.035 and 20.0% vs. 4.29%, *p* = 0.013, respectively). No significant differences in laboratory test results between the two subgroups were observed and heart rhythm types in ECG performed on discharge were similar. Occurrence of left bundle branch block (LBBB) or right bundle branch block (RBBB) by ECG on discharge was more frequent in event-positive subgroup (22.2% vs. 4.41%, *p* = 0.009), with a similar result for ST-segment depression (25.0% vs. 8.82%, *p* = 0.035). The Gensini score was higher in the event-positive group compared to the control group (11.2 ± 9.33 points vs. 7.78 ± 5.77 points, *p* = 0.034) and patients from the event-positive subgroup had a thicker intraventricular septum (IVS) and left ventricular posterior wall (PW) by ECG on discharge (12.1 ± 2.39 mm vs. 10.8 ± 1.86 mm, *p* = 0.006 and 11.1 ± 2.39 mm vs. 9.93 ± 1.6 mm, *p* = 0.006, respectively).

Pharmacotherapy at discharge is presented in Table 2. In the group of patients without a primary composite endpoint, β-blockers, statins, and SAPT were used more frequently, while patients from the other group were more often treated with Ca-blockers, ACEI/ARB, DAPT, DAPT+OAC and OAC. However, differences between those groups did not reach statistical significance.

Clinical outcomes after 3 years are listed in Table 3.

### 3.1. Kaplan–Meier Survival Estimator

**Figure 2:**
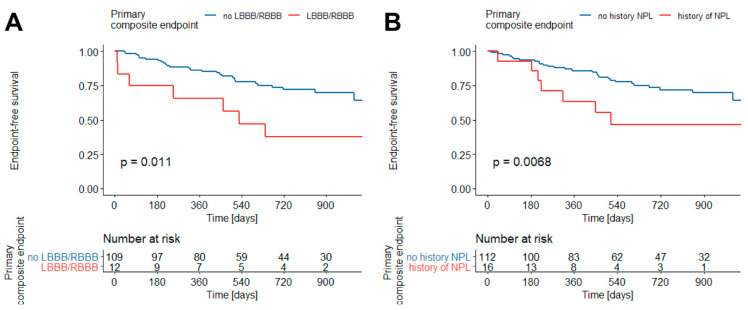
Primary composite endpoint free survival in patients with MINOCA and RBBB/LBBB in ECG on discharge (**A**) and history of neoplasm (**B**) (Kaplan–Meier estimator). Abbreviations: RBBB—right bundle branch block, LBBB—left bundle branch block, NPL—neoplasm.

Kaplan–Meier survival estimators indicated that primary composite endpoint-free survival is worse in patients with LBBB or RBBB present in ECG on discharge compared to patients without BBB (*p* = 0.011) (Figure 2A) and patients with a history of neoplasm in comparison to those with no prior neoplasm (*p* = 0.0068) (Figure 2B).

### 3.2. The Univariate Cox Proportional Hazards Model Analysis

**Figure 3:**
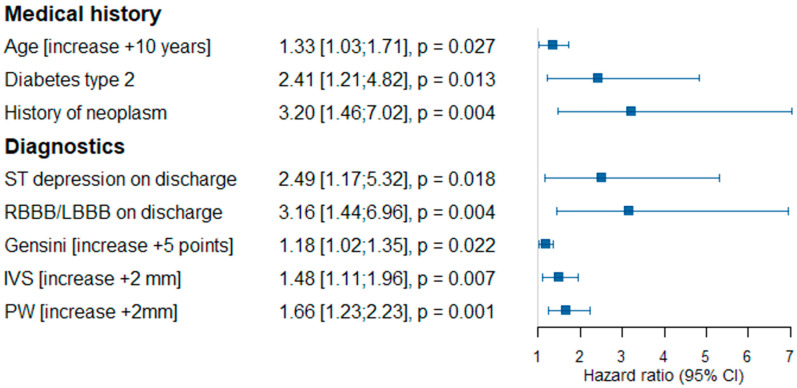
The univariate Cox proportional hazards model for primary composite endpoint occurrence. Abbreviations: HR—hazard ratio, RBBB—right bundle branch block, LBBB—left bundle branch block, IVS—intraventricular septum thickness, PW—posterior wall thickness.

The univariate Cox proportional hazards model analysis was made with the aim of determining the variables associated with primary composite endpoint occurrence (Figure 3). Age (additional 10 years; HR 1.33; 95% CI 1.03–1.71, *p* = 0.027), type 2 diabetes (HR 2.41; 95% CI 1.21–4.82, *p* = 0.013) and a history of neoplasm (HR 3.2; 95% CI 1.46–7.02, *p* = 0.004) were independently associated with primary composite endpoint occurrence. Furthermore, ST-segment depression by ECG on discharge (HR 2.49; 95% CI 1.17–5.32, *p* = 0.018), RBBB/LBBB by ECG on discharge (HR 3.16; 95% CI 1.44–6.96, *p* = 0.004), Gensini score (HR 1.18; 95% CI 1.02–1.35, *p* = 0.022), IVS thickness (additional 2 mm; HR 1.48; 95% CI 1.11–1.96, *p* = 0.007) and PW thickness (additional 2 mm; HR 1.66; 95% CI 1.23–2.23, *p* = 0.001) were diagnostic results found to be independent risk factors for primary composite endpoint occurrence.

### 3.3. The Multivariate Cox Proportional Hazards Model

**Figure 4:**
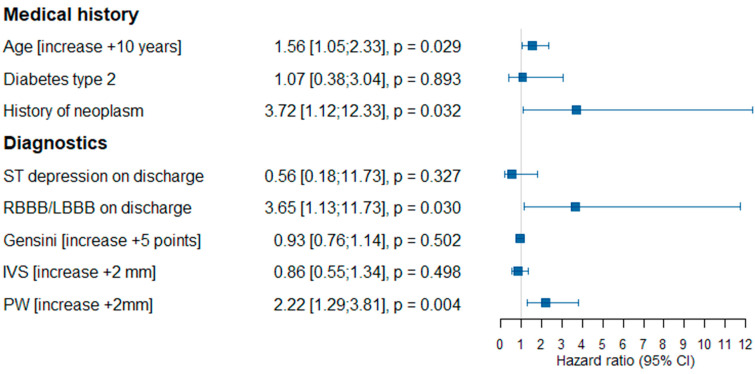
The multivariate Cox proportional hazards model for primary composite endpoint occurrence. Abbreviations: HR—hazard ratio, RBBB—right bundle branch block, LBBB—left bundle branch block, IVS—intraventricular septum thickness, PW—posterior wall thickness.

Using the multivariate Cox proportional hazards model, we confirmed four variables as risk factors of primary composite endpoint occurrence, namely age (additional 10 years; HR 1.56; 95% CI 1.05–2.33, *p* = 0.027), a history of neoplasm (HR 3.72; 95% CI 1.12–12.33, *p* = 0.032), RBBB/LBBB in ECG on discharge (HR 3.65; 95% CI 1.13–11.73, *p* = 0.030) and PW thickness (additional 2 mm; HR 2.22; 95% CI 1.29–3.81, *p* = 0.004) (Figure 4).

## 4. Discussion

The main findings from our study demonstrate that older age, diabetes, a history of neoplasm, ST-segment depression and RBBB/LBBB in ECG on discharge, higher Gensini score and higher thickness of IVS and PW are associated with worse long-term outcomes in patients presenting with MINOCA.

We enrolled all patients diagnosed with MINOCA in the mentioned time period, initially blinded for a clinical outcome to minimize the risk of selection bias. We gathered all available follow-up data to reduce information bias.

Diagnosis and treatment of patients who present with symptoms of MI and non-obstructive coronary arteries remain an unresolved clinical issue. Recent data demonstrated that no significant stenosis was found by coronary angiography in 10–20% of patients presenting with MI [4,13,14,15]. This phenomenon raised important but unanswered questions regarding the pathogenesis, diagnosis and treatment of MINOCA. The rate of adverse cardiovascular events and death is comparable between MINOCA and MI with obstructive coronary arteries [16]. In an analysis of approximately 2700 patients, Safdar et al. showed that patients with MINOCA and patients with AMI had comparable one-year mortality [17]. Kang et al. demonstrated a similar 12-month MACE rate in patients with MINOCA compared to patients with MI and either single or double-vessel CAD [18]. We observed relatively low mortality (13%) in MINOCA patients during a 3-year follow-up in a large retrospective study [5].

A recent multi-center cohort study of 16,849 MINOCA patients reported the rate of MACE events over 12 months at 18.7%, and one out of every five MINOCA patients suffered a major adverse event [19]. Therefore, additional risk stratification to further refine new clinical predictive factors is essential to identify MINOCA patients who are at increased risk of new MACE events.

The presented results suggest age, type 2 diabetes, a history of neoplasm, ST-segment depression and LBBB/RBBB by ECG on discharge, and increased thickness of IVS and PW as risk factors for worse long-term outcomes in patients with MINOCA.

In the 3-year follow-up, we observed approximately 36% of primary endpoint occurrence.

In a recent study, diabetes and age were shown as risk factors for ACS re-admission in patients with MINOCA [20]. Patients with diabetes and non-obstructive coronary artery disease can be characterized with a comparable risk of MI occurrence in ten years compared to the general population [21]. Moreover, Paolisso et al. indicated that type 2 diabetes in MINOCA patients results in higher death and MACE rate [22].

Active cancer was observed as a risk factor for MINOCA occurrence compared to patients with MI and obstructive coronary artery disease [23]. In a recent study, a history of former or active cancer was associated with a more frequent Takotsubo syndrome diagnosis. Moreover, diagnosis of MINOCA should be an indication for further investigation for the presence of an occult malignancy [24], potentially caused by thromboembolism, which is often suggested as an important pathomechanism of this process. These observations might be an explanation for the high rate of all-cause mortality observed in our registry as well.

In a large cohort study, Bansilal et al. assessed long-term outcomes in patients with RBBB, LBBB and no BBB registered on the initial ECG [25]. In that study, during the 7-year observation, MACE occurrence in patients with LBBB and RBBB was similarly higher in comparison to those without BBB. In this period, patients with RBBB were more likely to undergo pacemaker implantation. Both RBBB and LBBB groups were more likely to develop heart failure. In nearly 17-year observations of patients with RBBB and LBBB, patients had a higher mortality rate than those without BBB. However, after adjustment for risk factors and mortality only risk associated with LBBB remained significant [25]. To the best of our knowledge, a connection between BBB and worse outcomes in patients with MINOCA has not yet been described in the literature.

Although MINOCA patients do not have significant stenosis in coronary arteries by definition, they may present with insignificant coronary artery plaques. Therefore, 38% of patients with MINOCA still had observable plaque rupture and ulceration [16]. Ciliberti et al. observed that patients presenting with MINOCA and mild coronary artery disease (stenoses ≥30% but <50%) of three vessels or left main coronary artery characterized with worse long-term clinical outcomes in comparison to a mild coronary artery of one or two vessels [26]. Moreover, atherosclerosis may affect the whole vascular system and cause cardiovascular adverse events as a result [27]. There is scant literature concerning the extent of atherosclerotic plaques in coronary arteries characterized by the Gensini Score, and its impact on the clinical outcomes for MINOCA patients is therefore limited. Eskerund et al. demonstrated that left ventricle hypertrophy is an independent risk factor for both the occurrence and extent of MI in a group of MINOCA patients [28].

ST-segment elevation or depression by ECG at admission substantially modifies the management of patients presenting with ACS. Pelliccia et al. presented a meta-analysis including nearly 37,000 patients and indicates that ST-segment depression in ECG on admission is related to worse outcomes [1]. There is not enough data in the literature about the impact of ST changes in ECG on discharge on long-term prognosis in patients with MINOCA.

In a MicroCAD study, Eskerund et al. presented that left ventricular hypertrophy (LVH) expressed by increased thickness of IVS and PW is more common in patients with CAD and non-obstructive coronary arteries [28]. Furthermore, another study that analyzed patients with STEMI stated that LVH is associated with larger infarct size and worse outcomes in comparison to patients with STEMI and no LVH [29].

In our study, patients presenting with MINOCA were 3.7% of all acute myocardial infarction patients. However, this is significantly fewer than MINOCA occurrences published in recent articles [4,13,14,15]. The data for this study were assembled after the first mention of the MINOCA phenomenon, as released in the ESC guidelines on Management of Acute Myocardial Infarction in Patients Presenting with ST-Segment Elevation [8]. The current majority of investigations are based on data captured after the actualization of the MINOCA strategy in 2020 [9]. This appears to be the main reason for this discrepancy.

Treatment and secondary prevention in patients diagnosed with MINOCA is a key issue since this group can be characterized by a long-term prognosis compared to patients with MI and obstructive coronary artery disease. It was shown that ACEI and statins reduce the risk of long-term adverse event occurrences in patients with a history of MINOCA [5,30]. Furthermore, β-blockers seemed to improve the long-term prognosis for MINOCA patients [5,31]. Conversely, double antiplatelet therapy did not substantially change the prognosis in this group [5,30]. The lack of significant impact of pharmacological therapy on long-term outcomes observed in our registry might be an expression of the small sample analyzed.

Patients with a working diagnosis of MINOCA appear to be a strongly heterogeneous group. Conducting complex diagnostics (i.e., intravascular imaging, testing for coronary vasospasm and coronary microcirculation disorder, cardiac magnetic resonance imaging, transesophageal echocardiography, 24 h ECG registration) in these patients can lead to multiple details of the final diagnosis. Analysis of risk factors that will produce worse patient outcomes and specific treatments for the underlying cause of MINOCA is expected to reduce the rate of cardiovascular adverse events and cardiovascular readmissions. This detailed diagnostic and treatment process is especially important during the global COVID-19 pandemic when national healthcare systems suffer from outnumbered needs for hospitalization [32]. Re-admission in those conditions may meet difficulties and finally lead to extended numbers of severe complications including deaths.

### Study Limitations

This was a single-center study, the retrospective nature of which limited the amount and quality of the data gathered for analysis. However, consecutive patients were enrolled to generate representative clinical data. Long-term clinical follow-up of all patients was also not available. Most importantly, the study was carried out in a period where no clear algorithm for MINOCA assessment was established. The data in this study were gathered before 2020 and guidelines for the management of acute coronary syndromes without persistent ST-segment elevation including the actualized approach in MINOCA had not been outlined yet [9]. Application of the newest guidelines in MINOCA would result in conducting more complex diagnostics (i.e., intravascular imaging, testing for coronary vasospasm and coronary microcirculation disorder, cardiac magnetic resonance imaging, transesophageal echocardiography, 24 h ECG registration).

## 5. Conclusions

Our study revealed several factors associated with worse long-term outcomes in patients presenting with MINOCA, including typical cardiovascular risk factors such as older age and type 2 diabetes which in consequence may lead to the development of atherosclerotic lesions characterized by the Gensini score. What is more, myocardium hypertrophy, expressed by an increased intraventricular septum and posterior wall thickness which may be associated with the presence of RBBB, LBBB or ST-segment depression in ECG, was also identified as one of those risk factors. History of neoplasm may also have contributed to worse outcomes in MINOCA patients.

## Figures and Tables

**Table 1:** Baseline characteristics of the whole study group. Comparison between subgroups divided based on primary composite endpoint occurrence (myocardial infarction, percutaneous coronary intervention, stroke or TIA, all-cause death, re-admission due to cardiovascular event).

	Study Group	Primary Composite Endpoint		*p* Value
		No	Yes	
	n = 110	n = 70 (63.7%)	n = 40 (36.3%)	
Sex, male n (%)	42 (38.2%)	27 (38.6%)	15 (37.5%)	0.917
Age	64.9 (13.5)	62.9 (13.4)	68.4 (13.3)	0.040 *
BMI	27.7 (5.44)	27.5 (5.04)	28.2 (6.27)	0.607
Follow-up duration (days)	724 (308)	725 (305)	721 (317)	0.939
Medical history:				
Arterial hypertension	76 (69.1%)	44 (62.9%)	32 (80.0%)	0.064
Dyslipidemia	58 (52.7%)	34 (48.6%)	24 (60.0%)	0.257
Diabetes type 2	21 (19.1%)	9 (12.9%)	12 (30.0%)	0.035 *
Smoking	24 (21.8%)	16 (22.9%)	8 (20.0%)	0.743
Neoplasm	11 (10.0%)	3 (4.29%)	8 (20.0%)	0.013 *
Prior ACS	11 (10.0%)	4 (5.71%)	7 (17.5%)	0.063
Laboratory tests:				
Peak hsT troponin	0.48 (0.59)	0.53 (0.66)	0.39 (0.42)	0.238
Peak CKMB	38.2 (30.0)	40.9 (33.1)	33.5 (23.4)	0.230
WBC	9.59 (4.06)	9.74 (4.15)	9.32 (3.94)	0.595
HGB	13.6 (1.91)	13.8 (2.14)	13.4 (1.42)	0.324
LDL	2.85 (1.08)	2.86 (1.06)	2.83 (1.14)	0.880
CRP	13.2 (42.5)	16.8 (53.1)	7.42 (13.3)	0.399
D-dimer	1399 (2198)	1116 (1340)	1977 (3298)	0.180
eGFR	76.5 (21.5)	79.4 (22.0)	71.3 (19.9)	0.061
Coronary angiography:				
Gensini score	9.02 (7.41)	7.78 (5.77)	11.2 (9.33)	0.034 *
ECG on discharge				
Rhythm:				
Sinus	102 (92.7%)	67 (95.7%)	35 (87.5%)	0.242
AF	5 (4.55%)	2 (2.86%)	3 (7.50%)	
Stimulation	3 (2.73%)	1 (1.43%)	3 (5.00%)	
ST depression	15 (14.4%)	6 (8.82%)	9 (25.0%)	0.035 *
Reversed T wave	56 (53.8%)	33 (48.5%)	23 (63.9%)	0.142
LBBB/RBBB	11 (10.6%)	3 (4.41%)	8 (22.2%)	0.009 *
Echocardiography on discharge:				
IVS	11.2 (2.14)	10.8 (1.86)	12.1 (2.39)	0.006 *
PW	10.3 (1.98)	9.93 (1.60)	11.1 (2.39)	0.006 *
LVEF	54.2 (11.4)	54.9 (11.4)	53.1 (11.5)	0.441

Abbreviations: BMI—body mass index; ACS—acute coronary syndrome; CKMB—creatinine kinase MB isoenzyme; WBC—white blood count; HGB—hemoglobin; LDL—low-density lipoprotein; CRP—C-reactive protein; eGFR—estimated glomerular filtration rate; ECG—electrocardiography; AF—atrial fibrillation; LBBB—left bundle branch block; RBBB—right bundle branch block; IVS—intraventricular septum; PW—posterior wall; LVEF—left ventricle ejection fraction; *—statistical significance.

**Table 2:** Pharmacological treatment at discharge in the study group. Comparison between subgroups divided based on primary composite endpoint occurrence.

.	Study Group	Primary Composite Endpoint		*p* Value
		No	Yes	
	n = 110	n = 70 (63.7%)	n = 40 (36.3%)	
Pharmacological treatment:				
B-blocker	81 (73.6%)	54 (77.1%)	27 (67.5%)	0.282
Ca-blocker	39 (35.5%)	24 (34.3%)	15 (37.5%)	0.737
ACEI/ARB	88 (80%)	54 (77.1%)	34 (85.0%)	0.337
Statin	98 (89.1%)	64 (91.4%)	34 (85.0%)	0.321
SAPT	31 (28.2%)	23 (32.9%)	8 (20.0%)	0.156
DAPT	48 (43.6%)	29 (41.4%)	19 (47.5%)	0.544
DAPT+OAC	10 (9.1%)	6 (8.6%)	4 (10.0%)	0.798
OAC	16 (14.5%)	8 (11.4%)	8 (20.0%)	0.239

Abbreviations: B-blocker—beta blocker; Ca blocker—calcium channel blocker; ACEI—angiotensin-converting enzyme inhibitor; ARB—angiotensin receptor blocker; SAPT—single antiplatelet therapy; DAPT—double antiplatelet therapy; DAPT+OAC—double antiplatelet therapy+oral anticoagulant; OAC—oral anticoagulant.

**Table 3:** Occurrence of elements of the primary composite endpoint at 3-year follow-up.

Composite Endpoint	40 (36.4%)
Re-hospitalization due to CVD	16 (14.6%)
All-cause death	13 (11.8%)
Re-MI	7 (6.4%)
Stroke/TIA	3 (2.7%)
Revascularization (PCI/CABG)	1 (0.9%)

Abbreviations: ACS—acute coronary syndrome; TIA—temporary ischemic attack; PCI—percutaneous coronary intervention; CABG—coronary artery bypass graft; CVD—cardiovascular diseases; MI—myocardial infarction.

## Data Availability

The data presented in this study are available on request from the corresponding author.

## References

[1] Pelliccia F., Marzilli M., Boden W.E., Camici P.G. (2021). Why the Term MINOCA Does Not Provide Conceptual Clarity for Actionable Decision-Making in Patients with Myocardial Infarction with No Obstructive Coronary Artery Disease. J. Clin. Med..

[2] Tamis-Holland J.E., Jneid H., Reynolds H.R., Agewall S., Brilakis E.S., Brown T.M., Lerman A., Cushman M., Kumbhani D.J., Arslanian-Engoren C. (2019). Contemporary Diagnosis and Management of Patients with Myocardial Infarction in the Absence of Obstructive Coronary Artery Disease: A Scientific Statement from the American Heart Association. Circulation.

[3] Niccoli G., Camici P.G. (2020). Myocardial infarction with non-obstructive coronary arteries: What is the prognosis?. Eur. Heart. J. Suppl..

[4] Dees D., Rahimi F., Amann M., Nührenberg T.G., Löffelhardt N., Schmitz R., Valina C.M., Neumann F.-J., Hochholzer W. (2021). Prevalence and Causes of Myocardial Infarction with Non-Obstructive Coronary Arteries in a Contemporary Cohort of Patients with Suspected Myocardial Infarction. J. Clin. Med..

[5] Lindahl B., Baron T., Erlinge D., Hadziosmanovic N., Nordenskjöld A., Gard A., Jernberg T. (2017). Medical Therapy for Secondary Prevention and Long-Term Outcome in Patients with Myocardial Infarction with Nonobstructive Coronary Artery Disease. Circulation.

[6] Agewall S., Beltrame J.F., Reynolds H.R., Niessner A., Rosano G., Caforio A.L.P., De Caterina R., Zimarino M., Roffi M., Kjeldsen K. (2016). ESC working group position paper on myocardial infarction with non-obstructive coronary arteries. Eur. Heart J..

[7] Bryniarski K., Gasior P., Legutko J., Makowicz D., Kedziora A., Szolc P., Bryniarski L., Kleczynski P., Jang I.-K. (2021). OCT Findings in MINOCA. J. Clin. Med..

[8] Ibanez B., James S., Agewall S., Antunes M.J., Bucciarelli-Ducci C., Bueno H., Caforio A.L., Crea F., Goudevenos J.A., Halvorsen S. (2018). 2017 ESC Guidelines for the management of acute myocardial infarction in patients presenting with ST-segment elevation: The Task Force for the management of acute myocardial infarction in patients presenting with ST-segment elevation of the European Society of Cardiology (ESC). Eur. Heart J..

[9] Collet J.-P., Thiele H., Barbato E., Barthélémy O., Bauersachs J., Bhatt D.L., Dendale P., Dorobantu M., Edvardsen T., Folliguet T. (2021). 2020 ESC Guidelines for the management of acute coronary syndromes in patients presenting without persistent ST-segment elevation: The Task Force for the management of acute coronary syndromes in patients presenting without persistent ST-segment elevation of the European Society of Cardiology (ESC). Eur. Heart J..

[10] Thygesen K., Alpert J.S., Jaffe A.S., Chaitman B.R., Bax J.J., Morrow D.A., White H.D., Executive Group on behalf of the Joint European Society of Cardiology (ESC)/American College of Cardiology (ACC)/American Heart Association (AHA)/World Heart Federation (WHF) Task Force for the Universal Definition of Myocardial Infarction (2018). Fourth Universal Definition of Myocardial Infarction (2018). Circulation.

[11] Lang R.M., Badano L.P., Victor M.A., Afilalo J., Armstrong A., Ernande L., Flachskampf F.A., Foster E., Goldstein S.A., Kuznetsova T. (2015). Recommendations for cardiac chamber quantification by echocardiography in adults: An update from the American Society of Echocardiography and the European Association of Cardiovascular Imaging. J. Am. Soc. Echocardiogr..

[12] Gensini G.G. (1983). A more meaningful scoring system for determining the severity of coronary heart disease. Am. J. Cardiol..

[13] Larsen A.I., Galbraith P.D., Ghali W.A., Norris C.M., Graham M.M., Knudtson M.L. (2005). Characteristics and outcomes of patients with acute myocardial infarction and angiographically normal coronary arteries. Am. J. Cardiol..

[14] Gehrie E.R., Reynolds H.R., Chen A.Y., Neelon B.H., Roe M.T., Gibler W.B., Ohman E.M., Newby L.K., Peterson E.D., Hochman J.S. (2009). Characterization and outcomes of women and men with non-ST-segment elevation myocardial infarction and nonobstructive coronary artery disease: Results from the Can Rapid Risk Stratification of Unstable Angina Patients Suppress Adverse Outcomes with Early Implementation of the ACC/AHA Guidelines (CRUSADE) quality improvement initiative. Am. Heart J..

[15] Rakowski T., De Luca G., Siudak Z., Plens K., Dziewierz A., Kleczyński P., Tokarek T., Węgiel M., Sadowski M., Dudek D. (2019). Characteristics of patients presenting with myocardial infarction with non-obstructive coronary arteries (MINOCA) in Poland: Data from the ORPKI national registry. J. Thromb. Thrombolysis.

[16] Pasupathy S., Lindahl B., Litwin P., Tavella R., Williams M.J.A., Air T., Zeitz C., Smilowitz N.R., Reynolds H.R., Eggers K.M. (2021). Survival in Patients with Suspected Myocardial Infarction with Nonobstructive Coronary Arteries: A Comprehensive Systematic Review and Meta-Analysis from the MINOCA Global Collaboration. Circ. Cardiovasc. Qual. Outcomes.

[17] Safdar B., Spatz E.S., Dreyer R.P., Beltrame J.F., Lichtman J.H., Spertus J.A., Reynolds H.R., Geda M., Bueno H., Dziura J.D. (2018). Presentation, clinical profile, and prognosis of young patients with myocardial infarction with nonobstructive coronary arteries (MINOCA): Results from the VIRGO study. J. Am. Heart Assoc..

[18] Kang W.Y., Jeong M.H., Ahn Y.K., Kim J.H., Chae S.C., Kim Y.J., Hur S.H., Seong I.W., Hong T.J., Choi D.H. (2011). Are patients with angiographically near-normal coronary arteries who present as acute myocardial infarction actually safe?. Int. J. Cardiol..

[19] Dreyer R.P., Tavella R., Curtis J.P., Wang Y., Pauspathy S., Messenger J., Rumsfeld J.S., Maddox T.M., Krumholz H.M., Spertus J.A. (2020). Myocardial infarction with non-obstructive coronary arteries as compared with myocardial infarction and obstructive coronary disease: Outcomes in a Medicare population. Eur. Heart J..

[20] Nordenskjöld A.M., Lagerqvist B., Baron T., Jernberg T., Hadziosmanovic N., Reynolds H.R., Tornvall P., Lindahl B. (2019). Reinfarction in Patients with Myocardial Infarction with Nonobstructive Coronary Arteries (MINOCA): Coronary Findings and Prognosis. Am. J. Med..

[21] Olesen K.K.W., Madsen M., Gyldenkerne C., Thrane P.G., Thim T., Jensen L.O., Bøtker H.E., Sørensen H.T., Maeng M. (2021). Ten-year cardiovascular risk in diabetes patients without obstructive coronary artery disease: A retrospective Western Denmark cohort study. Cardiovasc. Diabetol..

[22] Paolisso P., Donati F., Bergamaschi L., Toniolo S., D’Angelo E., Magnani I., Angeli F., Bartoli L., Stefanizzi A., Foa’ A. (2020). Impact of type 2 diabetes mellitus and blood glucose admission levels in patients with myocardial infarction with non obstructive coronary artery disease (MINOCA). Eur. Heart J..

[23] Lopez-Pais J., Coronel B.I., Gil D.G., Jesús M., Pascual E., Durán B.A., Peredo C.G.M., Vinués C.M., García P.A., Gonzalez-Juanatey J.R. (2020). Clinical characteristics and prognosis of myocardial infarction with non-obstructive coronary arteries (MINOCA): A prospective single-center study. Cardiol. J..

[24] Kobo O.M., Vainer Evgrafov E., Cohen Y., Lerner Y., Khatib A., Hoffman R., Roguin A., Tzoran I. (2019). Non-ST-Elevation Myocardial Infarction with Non-significant Coronary Artery Disease as a Symptom of Occult or New Malignancy—PubMed. Isr. Med. Assoc. J..

[25] Bansilal S., Aneja A., Mathew V., Reeder G.S., Smars P.A., Lennon R.J., Wiste H.J., Traverse K., Farkouh M.E. (2011). Long-term cardiovascular outcomes in patients with angina pectoris presenting with bundle branch block. Am. J. Cardiol..

[26] Ciliberti G., Coiro S., Tritto I., Benedetti M., Guerra F., Del Pinto M., Finocchiaro G., Cavallini C., Capucci A., Kaski J.C. (2018). Predictors of poor clinical outcomes in patients with acute myocardial infarction and non-obstructed coronary arteries (MINOCA). Int. J. Cardiol..

[27] Miao B., Hernandez A.V., Alberts M.J., Mangiafico N., Roman Y.M., Coleman C.I. (2020). Incidence and Predictors of Major Adverse Cardiovascular Events in Patients with Established Atherosclerotic Disease or Multiple Risk Factors. J. Am. Heart Assoc..

[28] Eskerud I., Gerdts E., Larsen T.H., Lønnebakken M.T. (2019). Left ventricular hypertrophy contributes to Myocardial Ischemia in Non-obstructive Coronary Artery Disease (the MicroCAD study). Int. J. Cardiol..

[29] Stiermaier T., Pöss J., Eitel C., de Waha S., Fuernau G., Desch S., Thiele H., Eitel I. (2018). Impact of left ventricular hypertrophy on myocardial injury in patients with ST-segment elevation myocardial infarction. Clin. Res. Cardiol..

[30] Paolisso P., Bergamaschi L., Saturi G., D’Angelo E.C., Magnani I., Toniolo S., Stefanizzi A., Rinaldi A., Bartoli L., Angeli F. (2020). Secondary prevention medical therapy and outcomes in patients with myocardial infarction with non-obstructive coronary artery disease. Front. Pharmacol..

[31] Ciliberti G., Verdoia M., Merlo M., Zilio F., Vatrano M., Bianco F., Mancone M., Zaffalon D., Bonci A., Boscutti A. (2021). Pharmacological therapy for the prevention of cardiovascular events in patients with myocardial infarction with non-obstructed coronary arteries (MINOCA): Insights from a multicentre national registry. Int. J. Cardiol..

[32] Legutko J., Niewiara L., Bartus S., Dobrzycki S., Gasior M., Gierlotka M., Kochman J., Lesiak M., Matysek J., Ochała A. (2020). Decline in the number of coronary angiography and percutaneous coronary intervention procedures in patients with acute myocardial infarction in Poland during the coronavirus disease 2019 pandemic. Kardiol. Pol..

